# Successful Aging Perception in Middle-Aged Korean Men: A Q Methodology Approach

**DOI:** 10.3390/ijerph18063095

**Published:** 2021-03-17

**Authors:** Mi Hyeon Seong, Eunyoung Shin, Sohyune Sok

**Affiliations:** 1 Department of Nursing, Chang Shin University, Changwon 51352, Korea; mihyeon0624@cs.ac.kr; 2 Department of Nursing, Graduate School, Kyung Hee University, Seoul 02447, Korea; eyshin@khu.ac.kr; 3 Department of Nursing, College of Nursing Science, Kyung Hee University, Seoul 02447, Korea

**Keywords:** successful aging, middle-aged men, mature, leisure

## Abstract

The purpose of this study is to identify the types of perception of successful aging in middle-aged men and to analyze and describe the characteristics of each type of successful aging perception of middle-aged men in South Korea. This study uses an exploratory study design, applying the Q methodology, which is a subjective research method. The participants were 25 middle-aged men (40 to 60 years old) living in C, Y, and B cities, which were P-samples that were judged to best reveal the successful aging of middle-aged men. In this study, principal component analysis of the PQ method program was used. The five perception types of successful aging among middle-aged men are Type 1 for the “leisure type”, Type 2 for the “mature type”, Type 3 for the “health-oriented type”, Type 4 for the “patriarchal type”, and Type 5 for the “family-centered type”. The mature type had the highest variance among the five types, and the leisure type was the type that showed the second-highest variance. In nursing practice, nurses need to pay attention to the successful aging perceptions of middle-aged Korean men for their successful aging in the future.

## 1. Introduction 

In modern society, the human lifespan has increased due to the advancement of science and medicine, and demographic structure is shifting from an aged society to a super-aged society [1,2,3]. As a result of rising social welfare costs and increased expectations for old age, with, globally, 1.6 billion people aged 65 years or older by 2050, the successful aging of the elderly, which is a vulnerable group, is a major socioeconomic concern worldwide [3,4,5]. The concept of successful aging is widely used in the fields of society, mental society, and medicine and includes consideration of life satisfaction, well-being, maintenance of cognitive and physical functions, and mental and physical health [5,6]. In addition, successful aging means maintaining proper health during old age and the degree to which an individual maintains or recovers a high level of physical, mental, and social functions, which allow for a holistic assessment of the aging process [7].

Currently, most studies on successful aging are on elderly adults. There is a vulnerability that the process of healthy behavior is already underway and can be overlooked before aging [8]. In a recent study exploring successful aging, including that of middle-aged men, it was found that the level of successful aging was 19% lower than in previous studies [9]. These results suggest that intervention should be initiated from middle age as a predictive resource for successful aging [10]. For successful aging in old age, effective interventions against disability and health risks arising from aging are needed in order to lower morbidity and make the goal of aging plausible [4]. Since middle age is an important period that affects the lives and health of the elderly [11], future-oriented policies, measures, and programs, such as the revitalization of healthy cities that promote successful aging through self-integration from middle age and the development and dissemination of community-centered programs and old-age programs, are needed [4]. 

On the other hand, as the elderly generation rapidly loses their autonomy, various problems, including those related to welfare, are prevalent due to the increasingly elderly population [6]. Middle-aged men in South Korea perceive these problems as their future problems [12,13]. As they readily accept the reality of old age, going through a prosperous life and entering a period of many declines, middle-aged people are showing a strong desire to have a comfortable and healthy life during this stage [14]. Middle-aged men prioritize family support for their work life, and social retirement during this period acts as a serious event of losing status and authority as well as a stress factor [15]. It is difficult to have the time and mental space to establish new values and paradigms when these men are pushed out of their organizations without much time to prepare, unlike in developed countries that allow thinking and preparing for retirement [16].

Middle-aged men may experience a period of crisis while going through a major transition physically, economically, and socially in the middle of their life [17]. Compared to middle-aged women, the size of their social network and the degree of social support are less, so they face various difficulties [18]. In addition, middle-aged men have been overlooked because they are perceived to experience sudden biological events or developmental crises on physical, mental, social, and environmental levels compared to women [19]. For this reason, there is a need for in-depth research into successful aging and adaptation to an aging society that the current middle-aged men will face, preparation for long-term plans, and interventions for old age. Therefore, this study attempts to identify the phenomenon of successful aging, starting from the perspective of middle-aged male individuals through the Q methodology, which studies human subjectivity by focusing on a first-person perspective.

Q methodology is significant in that it creates a hypothesis through observation of the basis of hypothetical abduction, not hypothetical deduction, and evades standardized obtrusion caused by the investigator’s random and contrived questionnaires. It also demonstrates the hypothesizing feature of subjective thinking by depicting the mental state through spontaneous response and enables the discovery of the type formed by subjectivity structure initiated from one’s own viewpoint, not from the investigator’s hypothesis [20]. 

The purpose of this study is to apply Q methodology in exploring the types of subjectivity for successful aging, where middle-aged men are perceived subjectively, and the characteristics of each type in order to provide fundamental data for nursing intervention plans that can help middle-aged men adapt to the coming old age and improve their quality of life.

Accordingly, the aims of this study are to identify the types of perception of successful aging in middle-aged men and to analyze and describe the characteristics of each type of successful aging perception of middle-aged men.

## 2. Material and Methods

### 2.1. Study Design and Participants 

This study used an exploratory study design, applying Q methodology, which is a subjective research method, in order to systematically and scientifically explore the successful aging of middle-aged Korean men. Q methodology was created by William Stephenson in 1935. It is a method of systematically measuring subjective phenomena, such as opinions, attitudes, and values. It is a theoretical and methodological concept that increases the subjectivity of research participation or the first-person perspective [21]. This is based on the assumption of intrapersonal difference in meaning; by applying nonsmall numbers of individual differences, the importance of individual differences is seen. It is a useful research method for small sample analysis and audience analysis and is an appropriate method for objectively measuring the subjectivity of a specific subject [22]. The participants of this study were middle-aged men (40 to 60 years old) living in C, Y, and B cities, South Korea, who were P-samples that were considered the best for revealing the successful aging of middle-aged men in order to understand the types of perception of successful aging that middle-aged men subjectively perceive. A total of 25 people, including those who had already participated in the interview to form a Q-population, were conveniently sampled. Q methodology is a qualitative research study that emphasizes subjectivity as a method of studying subjective views on things that are personally or socially important [22,23]. This is not significantly limited by the P-sample; instead, when the P-sample becomes larger, there is a possibility that a statistical problem that cannot be clearly grasped due to the concentration of several people on one factor may occur. Therefore, the small sample doctrine and principle are followed, and the sampling method applied in social science research can be used [24].

### 2.2. Study Procedure

#### 2.2.1. Construction of Q-Population and Q-Sample

In this study, Q-population was constructed based on the data collected by reviewing the literature on successful aging and conducting in-depth interviews with middle-aged men. The subjects of in-depth interviews for obtaining the Q-population for the successful aging in middle-aged men were six middle-aged men (between 40 and 60 years old) who were healthy, could control themselves even though they had a disease, and could perform normal activities.

The in-depth interview about the perceptions of middle-aged men on successful aging was conducted for about 2 months, from March to April 2018, and 115 Q-populations were extracted through the in-depth interview process. Duplicate questions were deleted from the Q-populations extracted for the successful aging of middle-aged men, and two nursing professors participated together, in addition to the researcher, in order to readjust the sentences to a common meaning and value for each topic. There were 50 Q-samples selected through the process of exchanging opinions several times and revising whether each sentence was composed of statements that showed the subjectivity of the study participants (Table 1).

#### 2.2.2. P-Sample

Q-methodology is a method of studying subjective views on things that are personal or socially important; it is not greatly limited by the P-sample. Since there is a possibility that a statistical problem may occur, the small sample doctrine is followed [23].

This study is to understand the types of successful aging perception perceived subjectively by middle-aged adults. It is a P-sample that is judged to best reveal the successful aging of middle-aged adults. To form a recruitment group, 25 middle-aged men, including those who participated in the interview, were conveniently sampled.

#### 2.2.3. Q-Sort

**Figure 1:**
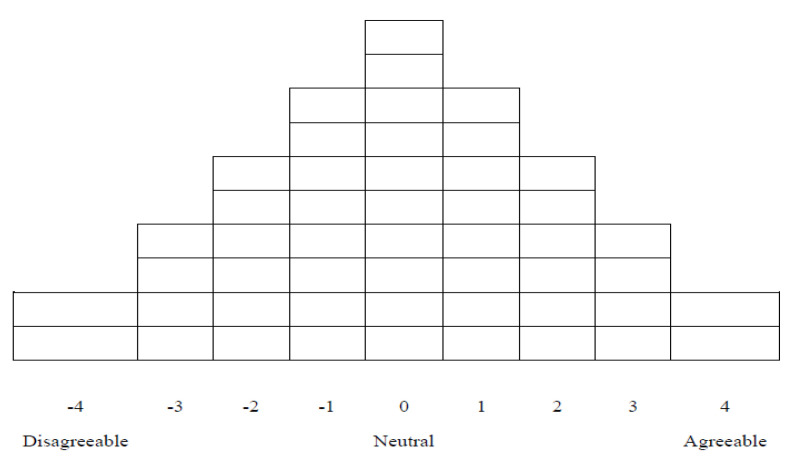
Card arrangement distribution table.

The time required for Q-sort, an investigation on general characteristics, and the interview was about 1 h, and the Q-sort period was about 3 months, from December 2018 to February 2019. For Q-sort, confirmed Q-samples were written on a paper card (Q-card), one by one, and numbered from 1 to 50, which is the last number. Each Q-statement card prepared in this way was read by the study participant and classified into three parts: agree (+), neutral (0), and disagree (−). After completing the classification into three groups, they read the statements of the group of Q-statement cards classified as “agree” and sorted the Q-statement cards again in order, starting from strongly agree (+4) to neutral (0). Similarly, the Q-statement card group classified as “disagree” was classified into strongly disagree (−4) to neutral (0) and arranged according to the card arrangement distribution table shown in Figure 1. After the classification was completed, efforts were made to obtain useful information for the interpretation of the Q-factor and to gain a broader understanding of the phenomenon through questions about the reason or feeling of classification and additional questions on successful aging for the statements placed on both ends. In order to ensure that the interview proceeded naturally, consent was obtained in advance to record the interview with a digital recorder and use it for the interpretation of the results. While the subject was performing the Q-sort, the researcher sat close to the subject, provided the subject with instructions on the sorting method, and collected data on general characteristics, such as age, gender, and educational attainment. The Q-sort, questionnaire preparation, and interview took about 30 min to 1 h, with an average of about 40 min.

### 2.3. Statistical Analysis 

In this study, principal component analysis of the PQ method program was used for the analysis. For the collected data, 50 Q-statements were entered according to the level of agreement or disagreement of each P-sample by assigning −4 points for the most disagreeable item, 0 points for the neutral item, and 4 points for the most agreeable item. In order to determine the ideal number of factors, an Eigenvalue of 1.0 or higher, which was determined to be the most appropriate, was selected. Next, for the analysis and naming of the Q-type, the reasons for the selection of agreed and disagreed items in each type and the characteristics of the type were comprehensively analyzed by considering the data on general characteristics, questionnaire data, and statements of the reasons for the items that showed strong agreement and strong disagreement when sorting the Q-samples. 

### 2.4. Ethical Considerations

The study was approved by the Institutional Research Board of K University (KHSIRB-18-003). Furthermore, the purpose and procedure of the study were explained to the subjects, and written informed consent for the study was received. Participation in the study was voluntary, and it was explained to the subjects that they could withdraw their intention to participate in the study at any time without consequences.

## 3. Results

In the result derived using the PQ method program, an Eigenvalue of the factor of 1.0 or more, the appropriate explanation of the factors for the total variance, and the uniqueness of each type, expressing the perception of successful aging in middle-aged men, should appear. It was classified into 5 types based on the method and analyzed.

### 3.1. Result Analysis 

In this study, 7 out of 25 subjects (P-1, -3, -6, -9, -15, -17, and -23) were not classified as one factor because the difference in factor weights was not significant. The factor score reflects a specific attitude, and the higher the weight, the closer to the axis (factor) of factor analysis. This also indicates that it is located farther from the origin. Therefore, the higher the factor weight, the more typical or representative the person of the factor is [22]. Age ranged from 40 to 58 years old. For religion, there were 11 Christians, 10 people with no religion, 3 Buddhists, and 1 Catholic (Table 2). The five types of perception on successful aging of middle-aged men in this study were classified based on the characteristics of each type: Type 1 was the “leisure type” (4 persons), Type 2 was the “mature type” (8 persons), Type 3 was the “health-oriented type” (3 persons), Type 4 was the “patriarchal type” (1 person), and Type 5 was the “family-centered type” (2 persons). The Eigenvalue for each type of this study is shown in Table 3, and the explanatory power was 64%. In the correlation between types, showing similarity by type, the correlation coefficient between Type 1 and Type 4 was r = 0.01, and the correlation coefficient between Type 4 and Type 5 was r = −0.02, which were both slightly low. The correlation coefficient between Type 1 and Type 2 was high at r = 0.63 (Table 4).

### 3.2. Q-Type Analysis

For the analysis, the characteristics of each type were described by focusing on the contents based on the type-specific score table, demographic characteristics, and in-depth interviews. The score for each type of statement is a standard score, which allows the first impressions of a factor to be interpreted by paying attention to items greater than ±1.00; demographic characteristics are used to estimate the unique properties of a factor. In addition, the in-depth interview data allow for the analysis of the factors through a three-dimensional understanding by observing their correlation in relation to the factors [22].

#### 3.2.1. Type 1: Leisure Type

Type 1 subjects perceive that successful aging indicates having hobbies, proper health maintenance, and financial support. They are characterized by living comfortably, enjoying life with a spouse, and placing importance on a life in which they can live harmoniously with young people. They also revealed that the spirit of challenge is important, not age, when it comes to having hobbies that they can enjoy after their retirement (Table 5). The following are meaningful statements explaining the reason why the respondents with the representativeness of Type 1 (factor weight of 1.0 or more) chose the extremes of the Q distribution table. Subject P-20 (factor weight = 0.74), who is the epitome of this type, said, *“After retirement, I think I must have at least one hobby like golf that I can enjoy with my spouse. A hobby like golf, wherein I can go out on a field, takes all day. All of our children are gone and we are lonely. If one person stays at home while the other goes out for a hobby, the couple will become more and more separated. I think that is when couples start to enjoy living separately and they consider going through a twilight divorce. After our children found each other’s mates, I only have my wife, and we have to be together.”* Subject P-18 (factor weight = 0.72) said, *“I went through hardships when I was young, so I have decided that after I retire, I will do everything I wanted to do. Age doesn’t matter. The will to do anything is important. With that in mind, I stood up during the IMF, and after retirement, I will enjoy my life. I am confident that I have enough money to spend on whatever I want.”*

Based on these results, Type 1, with these characteristics, was named the “leisure type” in this study.

#### 3.2.2. Type 2: Mature Type

Type 2 subjects tend to seek spiritual health and maturity. They are characterized by their inclination toward placing importance on maturity, insight, and health as they age, rather than the economic requirements for successful aging. In addition, it was found that Type 2 subjects prefer to maintain their mental health, live a mature life, and think more deeply about their retirement life for successful aging. They show a tendency to pursue a life of gaining the trust of the people around them and being considerate of others (Table 6). Subject P-2 (factor weight = 0.77), who represents Type 2, said, *“When I was young, there was a time when I lived immaturely. Now, as I am getting older, I have questions about why I did it in the past. However, I think that it would have been nice to do this at the time because I think that it has become the basis of where I am standing. When I get older, I would like to give advice and encourage young people so that they will not suffer from trial and error. I experienced great difficulty before achieving what I have now.”* Additionally, subject P-25 (factor weight = 0.66) said, *“Everyone goes to nursing hospitals when they get old these days. Until then, I want to live with dignity. Even if I get sick, it will only last for a month. I want to die while sleeping quietly without alarming my children. In order to do that, I have to plan ahead and get insurance as soon as possible.”*

Based on these results, Type 2, with these characteristics, was named the “mature type” in this study.

#### 3.2.3. Type 3: Health-Oriented Type

Type 3 subjects indicated that health should be prioritized for successful aging and that time should be given to life in their old age in order to take full care of themselves, freeing them from their responsibility as the head of the household. Although they are supported by their family members, they tend to think that it is natural as the head of the family to place importance on the benefits of interpersonal relationships. In addition, they think that successful aging is achieved if social success leads to life in old age (Table 7). Subject P-7 (factor weight = 0.68), who represents Type 3, said, *“Can we say that we have successfully grown old without prioritizing our health? I want to live without illness until the age of 100. I do not intend to benefit from my children, and my wife is still busy enjoying her life, so I have to take care of myself. I went through hardships. I do not want to go into a nursing hospital with a diaper if I get sick. I want to stay healthy as much as possible. Therefore, I have to exercise and take care of myself.”* Additionally, subject P-8 (factor weight = 0.67) said, *“I played the role of head of the household more than enough, so I have to focus on taking care of myself. I have been interested in nutritional supplements, which I did not take because they were unpleasant. When I get old, I want to meet my friends whenever I want, and I want to look after the people I have forgotten because I was busy. Now, I have a high position in the company and I am making money. However, when I retire, I am just an old person who still wants to be important to his family and to other people."*

Based on these results, Type 3, with these characteristics, was named the “health-oriented type” in this study.

#### 3.2.4. Type 4: Patriarchal Type

Type 4 subjects want their children to take care of them in return for their devotion to them when they were young. They also express the desire to live harmoniously with their families. The subjects of this type exhibit the characteristics of a typical head of a household but also show the characteristics of trying not to be marginalized by their families after their retirement (Table 8). Subject P-22 (factor weight = 0.89), who is the epitome of this type, said, *“I want my children to visit me often when I am old, as I did to my parents. Maybe I am inflexible. I want them to help me if I am sick, even though they do not care about me. Actually, I have been dedicated to providing for my family and raising my children. Is it too much to ask? I would like to boast that my children will take care of me even if I get old and sick. It would bring me so much joy. I would like to watch TV with my grown-up children on holidays. Kids these days have strong individualism, so I don’t know how many times a year I will be able to see them before I die. Families shouldn’t do that, but it worries me.”*

Based on these results, Type 4, with these characteristics, was named the “patriarchal type” in this study.

#### 3.2.5. Type 5: Family-Centered Type

Type 5 subjects value family relationships even in their old age and tend to feel responsible and committed to their families. They show a sense of responsibility as the head of the family, and they did not want to become a burden to their families, with characteristics showing anxiety about aging and being the head of the household. Due to their dedication to their families, they tend to neglect their interpersonal relationships. Nevertheless, they show a willingness to improve after retirement (Table 9). Subject P-19 (factor weight = 0.77), who is the epitome of this type, said, *“I only have my family. I have always lived for my family, and I have shared emotions with them. Even if I retire, I am still the head of the family. I hope that our family will be together forever. However, I do not want anything big. Instead, when my son marries, I want to buy him a house, and it worries me. Even if it is difficult, it would be all right if we help each other and make an effort. To be honest, I lived my life saving money without meeting my friends because I was dedicated to my family. There were times when it was not worth the money to meet a friend for a drink. Now, I wanted to meet them. Even so, I have a few friends who will be happy to meet me even if I contact them out of nowhere.”* Additionally, subject P-21 (factor weight = 0.57) said, *"Honestly, facing death is scary. I have hypertension, so I take blood pressure pills. However, I do not want to bother my children, so I am doing my best to exercise.”*

Based on these results, Type 5, with these characteristics, was named the “family-centered type” in this study.

## 4. Discussion

This study used Q methodology to identify the types of perception of successful aging in middle-aged men, and it explains the characteristics of each type. This provides fundamental data for the development of effective nursing intervention plans for successful aging among middle-aged men. Although successful aging has contributed to improving the potential and quality of life of older adults with prejudice toward aging, the criteria are overly ideal, marginalizing many people who do not meet the criteria for successful aging and having a negative impact on ego formation [25]. Therefore, this study attempted to provide a foothold for successful aging in old age by encouraging middle-aged men to prepare and think about their life in old age through a study on finding out and realizing their perceptions on successful aging. In this study, five perception types of successful aging among middle-aged men were confirmed: Type 1 for the leisure type, Type 2 for the mature type, Type 3 for the health-oriented type, Type 4 for the patriarchal type, and Type 5 for the family-centered type.

Type 1 (leisure type) is the type that shows the second-highest variance among the five types. Subjects of this type value leisure activities with their spouse and believe that economic power and health are necessary. The analysis result showed that the subjects of Type 1 recognize leisure activities as a very important requirement for successful aging, thus showing a tendency to achieve satisfaction through leisure activities after their retirement and pursue economic independence and the maintenance of health.

Type 2 (mature type) has the highest variance among the five types. Subjects of this type recognize mental health, mental maturity, and psychological happiness as requirements for successful aging. Among the three dimensions of Rowe and Kahn’s successful aging model, cognitive function is required, which is important for maintaining the autonomous life of the elderly. It is also explained as necessary for controlling themselves and maintaining activities of daily living. In this study, the subjects showed a strong agreement on mental maturity, with the idea that they want to find and develop psychological satisfaction on their own, thus going further from Rowe and Kahn’s successful aging conditions. 

Type 3 (health-oriented type) places importance on the health and function of the body for successful aging. Subjects of this type focus on living to a healthy old age. The subjects of Type 3 pointed out that health should be prioritized for successful aging and that they should be freed from their responsibilities as the head of the household by being providing sufficient care for their old life. Although they are supported by their families, they tend to take it for granted as the head of the family and value the benefits of interpersonal relationships. In addition, they generally think that successful aging is achieved if social success leads to life in old age.

Type 4 (patriarchal type) has a family-centered way of thinking, placing emphasis on interpersonal relationships with many people for successful aging. Subjects of this type have a tendency to want their children to take care of them in return for their devotion to their families. They also expressed the desire to live harmoniously with their families. Type 4 subjects exhibit the characteristics of a typical head of household, and they do not want to be alienated by their families after retirement.

Type 5 (family-centered type) has a family-centered way of thinking and values family relations. Subjects of this type tend to feel responsible and devote themselves to their families. Type 5 subjects have family-centered values; however, they had a tendency to neglect interpersonal relationships due to their family-centered way of thinking. However, they show a willingness to improve. They are slightly anxious about aging but acknowledge that proper healthcare is necessary for successful aging.

Among existing studies on successful aging in middle-aged men, most of them were quantitative studies that dealt with the effects of their work life, the relationship with their spouses, and the presence or absence of their spouses [13,16,26]. They also examined the factors affecting successful aging in elderly males [26]. However, no studies have been conducted on the different types of perception of successful aging in middle-aged men. In this study, the five types of perception of successful aging in middle-aged men represent various characteristics by attribute and dimension for successful aging in middle-aged men, compared to Rowe and Kahn’s successful aging model [27]. This study is thought to present the typical characteristics of successful aging in middle-aged men.

The study results show that middle-aged men value positive factors or interactions from their relationships with the people around them, including family relationships. This can be explained in terms of active life participation in the interpersonal relationship aspect of Rowe and Kahn’s model [27]. An interpersonal relationship is not a comprehensive relationship between individuals; instead, it refers to the formation of a relationship among individuals, such as the emotions between two individuals, their actions, and their expectations of other people [28]. In the case of old age, such interpersonal relationships will have been reduced due to the death of their spouse, brothers, sisters, or nuclear family [28,29]. Research also shows that the elderly who are actively involved in such interpersonal relationships have a higher level of successful aging than those who are passive [30]. Hong and Lim [31] said that middle-aged people tend to maintain the social relationships that they have built up at work and in society even after they become old and that people with high self-esteem tend to value interpersonal relationships. The results of these previous studies suggest that a nursing intervention strategy related to interpersonal relationships should be prepared for the successful aging of middle-aged men through active participation in life. In particular, the middle-aged men in this study responded sensitively to the Q questions related to social relationships and family. It is in line with the description that successful aging for them means that they want to spend their old age interacting with friends, spouse, family members, or surrounding people rather than spend time alone when they perform any activity or action. In addition, the Q-questions to which the middle-aged men showed strong agreement or strong disagreement in this study included interactions among people as well as the confidence and trust that they receive from others. In other words, it can be said that middle-aged men believe that the emotionally positive effects that they have given to others, and vice versa, are necessary for successful aging, even in interpersonal relationships. Based on this study and the previous studies, interpersonal relationships can be seen as an important variable for the successful aging of middle-aged men. It is, therefore, necessary to prepare intervention plans to include this. However, it is difficult to find studies on the effects of interpersonal relationships of middle-aged men on successful aging or studies on interpersonal relationships of middle-aged men. Based on this study, it is necessary to see what factors influence the interpersonal relationships of middle-aged men, what influences the subfactors have on the quality of life in old age, and if they are important requirements for successful aging.

Based on the above results, the successful aging perception types of middle-aged Korean men were confirmed, which are expected to improve the understanding of the subjective aspect of each type and help develop newly defined successful aging perception types of middle-aged men to lay the foundation for a new theoretical framework. Five types of successful perceptions of aging in middle-aged men were identified. These results support the opinions of previous studies where the family factor has an important influence on the life satisfaction of middle-aged men due to the family-centered values that middle-aged Korean men place meaning on in their lives, which are the health, harmony, and success of their children rather than themselves. It shows the reflected result and indicates that it can act as an important factor in life in old age [32,33,34]. 

Since this study was conducted on middle-aged men living in some cities, it is necessary to be careful when generalizing the study results. In addition, there is a limitation in extending the interpretation to all middle-aged men in South Korea. Therefore, it is necessary to repeat and expand the study in the future while taking into consideration the sampling of the subjects. Currently, studies on aging among middle-aged people worldwide, including Korea, are insufficient, and different results may be shown depending on demographic characteristics. In particular, Korean society is based on the Confucian culture, and various values exist due to Western culture. This study was able to confirm these sociocultural characteristics once again, and it is possible to explore various perspectives on aging in modern Korean society in the 21st century through the subjectivity of middle-aged men. Since the psychological aspects of successful aging can have a significant physical effect on aging, further study is deemed necessary.

## 5. Conclusions

In conclusion, it was found that there were differences in the values that middle-aged Korean men consider important or pursue based on each type of perception of successful aging. Accordingly, the viewpoint of successful aging may be different. In addition, middle-aged men accept aging as a natural process and recognize it as a controllable problem. Through this study, it is expected that the understanding of the subjective aspects of each type will be enhanced, and the newly defined successful aging perception patterns of middle-aged men can establish a new theoretical framework. It is expected that a plan will be prepared for middle-aged people to adapt to aging and achieve self-integration in their old age. In addition, it is expected that it can be used as basic data for the development of an effective nursing intervention plan for the successful aging perception of middle-aged men and the achievement of successful aging in a prolonged life of old age.

## Figures and Tables

**Table 1:** Fifty Q-statements.

1. I must be healthy enough to be active.
2. Traveling, working, or moving a lot due to exercise must be endurable.
3. I am reluctant to use my body a lot.
4. I have more wrinkles or gray hair than other people my age, and I am unable to manage them.
5. I shouldn’t ever have a major illness.
6. I have a chronic disease, but it must be managed without any problems.
7. There should be no problems with sexual function.
8. There should be no problems with urinary or fecal incontinence.
9. It seems odd that the body reacts sensitively to the weather.
10. I need to be in control of my emotions.
11. I am afraid of getting old.
12. There should be room for every business.
13. I think I’m getting older because I have a lot of medications to take.
14. There are many difficulties when interacting with the younger generation.
15. I should be able to respond flexibly when faced with difficult problems.
16. Aging cannot be prevented; however, it can be acknowledged and compromised.
17. There is a desired end of life, but I am not ready for it.
18. I have to create new hobbies in my old age and live without boredom.
19. Challenge is for the young people, and it’s more important to stay in our present life.
20. It is an aging process, and there are now limits to what seemed possible to do.
21. After retirement, I should be able to do what I couldn’t do when I was young.
22. I have a lot of regrets when I look back on the past.
23. I should feel like I’ve lived a life of effort and I am being rewarded.
24. I have to be more mature than when I was young.
25. I am not proud of myself.
26. There were beliefs and values that I was thinking about. However, as I get older, this is meaningless and my work becomes important.
27. There has to be someone I can meet at any time.
28. I am so busy that I cannot afford to take care of the people around me.
29. It is difficult to find a friend who can readily help me when I am in trouble.
30. As I get older, I should have more meetings (3 or more) and interact with many people.
31. I feel that I am not trusted by the people around me.
32. I should feel respected by people.
33. There is no need to call to say hello or visit anyone during holidays or special days.
34. When the people around me are facing difficulty, they look for me first.
35. My family is generally healthy.
36. My children grew up as I wished.
37. I should have a hobby or activity that I can enjoy with my spouse.
38. It is difficult to openly talk about what I am feeling with my family.
39. Our families should be united and able to eat often together.
40. Even in old age, there is a great burden of being responsible for my family.
41. To my family, I’m still an important person and I have family members to support.
42. I am not afraid of getting old or dying because of my family.
43. Even if I die, my family seems to have no problem.
44. When I’m old or sick, someone in my family should support me.
45. I should be prepared for old age.
46. When I want to go on a trip, I have to be able to go or have the money to do what I want.
47. Even after retirement, I must continue to do economic activities.
48. I have insurance in case of sickness, so I don’t worry too much about the state of my finances.
49. I don’t need to have any property to pass on to my children.
50. I can’t afford to help those in need.

**Table 2:** Factor weight and general characteristics of P-samples according to type (n = 25).

Type	P-Sample	Factor Weight	Age (Year)	Marital Status	Child (Ren)	Education	Religion	Job	Economic State
1	P-4	0.70	40	Married	Yes	University	Buddhism	Profession	High
(n = 4)	P-12	0.70	40	Married	Yes	Graduate school	None	Office worker	Moderate
	P-18	0.72	42	Married	Yes	University	None	Profession	High
	P-20	0.74 *	52	Married	Yes	University	None	Office worker	High
2	P-2	0.77 *	60	Married	Yes	Graduate school	Buddhism	Office worker	Low
(n = 8)	P-5	0.63	56	Married	Yes	University	None	Profession	High
	P-10	0.56	45	Married	Yes	University	Protestant	Public official	Moderate
	P-13	0.72	41	Married	Yes	University	None	Self-employed	High
	P-14	0.75	45	Married	Yes	University	Protestant	Other	Low
	P-16	0.71	58	Married	Yes	University	Protestant	Public official	Low
	P-24	0.53	47	Married	Yes	Graduate school	Protestant	Office worker	High
	P-25	0.66	48	Married	Yes	University	None	Office worker	High
3	P-7	0.68 *	40	Single	Yes	University	None	Public official	Low
(n = 3)	P-8	0.67	45	Married	Yes	University	Protestant	Public official	Moderate
	P-11	0.62	42	Married	Yes	University	None	Public official	Moderate
4 (n = 1)	P-22	0.89 *	57	Married	Yes	University	None	Office worker	Moderate
5	P-19	0.77	56	Married	Yes	University	Protestant	Office worker	High
(n = 2)	P-21	0.57	59	Married	Yes	University	Protestant	Public official	High

* Typical type.

**Table 3:** Eigen value and variance according to type.

Type	Type 1	Type 2	Type 3	Type 4	Type 5
Eigen values	4.16	5.80	2.84	1.44	2.01
Variance (%)	17	22	11	6	8
Cumulative (%)	17	39	50	56	64

**Table 4:** Correlations according to type.

Type	Type 1	Type 2	Type 3	Type 4	Type 5
Type 1	1.00				
Type 2	0.63	1.00			
Type 3	0.45	0.50	1.00		
Type 4	0.01	−0.04	−0.05	1.00	
Type 5	0.43	0.35	0.13	−0.02	1.00

**Table 5:** The Q-statements with strong agreement or disagreement by Type 1.

Agreement		Standard Score
1.	I must be healthy enough to be active.	2.03
46.	When I want to go on a trip, I have to be able to go or have the money to do what I want.	2.01
2.	Traveling, working, or moving a lot due to exercise must be endurable.	1.87
45.	I should be prepared for old age.	1.73
37.	I should have a hobby or activity that I can enjoy with my spouse.	1.54
35.	My family is generally healthy.	1.50
18.	I have to create new hobbies in my old age and live without boredom.	1.23
21.	After retirement, I should be able to do what I couldn’t do when I was young.	1.05
**Disagreement**		**Standard Score**
25.	I am not proud of myself.	−1.86
19.	Challenge is for the young people, and it’s more important to stay in our present life.	−1.40
22.	I have a lot of regrets when I look back on the past.	−1.40
49.	I don’t need to have any property to pass on to my children.	−1.37
11.	I am afraid of getting old.	−1.22
3.	I am reluctant to use my body a lot.	−1.21
31.	I feel that I am not trusted by the people around me.	−1.16
13.	I think I’m getting older because I have a lot of medications to take.	−1.11

**Table 6:** The Q-statements with strong agreement or disagreement by Type 2.

Agreement		Standard Score
1.	I must be healthy enough to be active.	2.37
12.	There should be room for every business.	1.87
10.	I need to be in control of my emotions.	1.79
24.	I have to be more mature than when I was young.	1.63
37.	I should have a hobby or activity that I can enjoy with my spouse.	1.39
15.	I should be able to respond flexibly when faced with difficult problems.	1.29
2.	Traveling, working, or moving a lot due to exercise must be endurable.	1.20
18.	I have to create new hobbies in my old age and live without boredom.	1.10
**Disagreement**		**Standard Score**
25.	I am not proud of myself.	−1.86
4.	I have more wrinkles or gray hair than other people my age, and I am unable to manage them.	−1.85
26.	There were beliefs and values that I was thinking about. However, as I get older, this is meaningless and my work becomes important.	−1.74
19.	Challenge is for the young people, and it’s more important to stay in our present life.	−1.63
11.	I am afraid of getting old.	−1.45
29.	It is difficult to find a friend who can readily help me when I am in trouble.	−1.46
31.	I feel that I am not trusted by the people around me.	−1.20
28.	I am so busy that I cannot afford to take care of and consider the people around me.	−1.06

**Table 7:** The Q-statements with strong agreement or disagreement by Type 3.

Agreement		Standard Score
1.	I must be healthy enough to be active.	2.13
8.	There should be no problems with urinary or fecal incontinence.	1.94
7.	There should be no problems with sexual function.	1.80
15.	I should be able to respond flexibly when faced with difficult problems.	1.57
5.	I shouldn’t have ever had a major illness.	1.48
18.	I have to create new hobbies in my old age and live without boredom.	1.48
2.	Traveling, working, or moving a lot due to exercise must be endurable.	1.39
41.	To my family, I’m still an important person and I have family members to support.	1.21
**Disagreement**		**Standard score**
29.	It is difficult to find a friend who can readily help me when I am in trouble.	−1.71
28.	I am so busy that I cannot afford to take care of and consider the people around me.	−1.71
44.	When I’m old or sick, someone in my family should support me and have someone like that.	−1.57
11.	I am afraid of getting old.	−1.57
19.	Challenge is for the young people, and it’s more important to stay in our present life.	−1.30

**Table 8:** The Q-statements with strong agreement or disagreement by Type 4.

Agreement		Standard Score
30.	As I get older, I should have more meetings (3 or more) and interact with many people.	1.98
50.	I can’t afford to be refreshed to help those in need.	1.98
43.	Even if I die, my family seems to have no problem.	1.49
44.	When I’m old or sick, someone in my family should support me.	1.49
45.	I should be prepared for old age.	1.49
49.	I don’t need to have any property to pass on to my children.	1.49
**Disagreement**		**Standard Score**
4.	I have more wrinkles or gray hair than other people my age, and I am unable to manage them.	−1.98
11.	I am afraid of getting old.	−1.98
14.	There are many difficulties when interacting with the younger generation.	−1.49
24.	I have to be more mature than when I was young.	−1.49
33.	There is no need to call to say hello or visit anyone during holidays or special days.	−1.49
13.	I think I’m getting older because I have a lot of medications to take.	−1.49

**Table 9:** The Q-statements with strong agreement or disagreement by Type 5.

Agreement		Standard Score
41.	To my family, I’m still an important person and I have family members to support.	2.30
45.	I should be prepared for old age.	1.94
30.	As I get older, I should have more meetings (3 or more) and interact with many people.	1.55
39.	Our families should be united and able to eat often together.	1.50
27.	There has to be someone I can meet at any time.	1.37
6.	I have a chronic disease, but it must be managed without any problems.	1.15
29.	It is difficult to find a friend who can readily help me when I am in trouble.	1.02
33.	There is no need to call to say hello or visit anyone during holidays or special days.	1.02
**Disagreement**		**Standard Score**
40.	Even in old age, there is a great burden of being responsible for my family.	−2.30
3.	I am reluctant to use my body a lot.	−2.12
49.	I don’t need to have any property to pass on to my children.	−1.55
22.	I have a lot of regrets when I look back on the past.	−1.37
8.	There should be no problems with urinary or fecal incontinence.	−1.19
44.	When I’m old or sick, someone in my family should support me.	−1.11
28.	I am so busy that I cannot afford to take care of and consider the people around me.	−1.02

## Data Availability

No new data were created or analyzed in this study. Data sharing is not applicable to this article.
